# Comparison between two triage scales requires testing users who do not have a known scale, with referent scenarios including all pathologies: Response letter to Miraghi about our study: FRENCH versus ESI: comparison between two nurse triage emergency scales with referent scenarios published in BMC Emergency Medicine: doi.org/10.1186/s12873-022-00752-z

**DOI:** 10.1186/s12873-023-00816-8

**Published:** 2023-05-24

**Authors:** Antoine Aubrion, Romain Clanet, Richard Macrez

**Affiliations:** 1 Department of emergency medicine, Caen-Normandie Hospital (CHU), Caen, France; 2 Emergency department, Bayeux Hospital, Bayeux, France; 3 Emergency department, Caen University Hospital, Caen, France; 4 Physiopathology and Imaging of Neurological Disorders, Normandie Univ, UNICAEN, INSERM, INSERM, UMR-S U1237, Institut Blood and Brain @ CaenNormandie, GIP Cyceron, Boulevard Becquerel, Caen, 14074 France

Dear Editor,

We thank Mirhaghi for their response letter, which helps us highlight and clarify several points of our work.

Our objective was to compare the direct validity of the French and ESI scales, by the rate of correct results in comparison to the expected result.

The specific cases used are those proposed by the experts who constructed the ESI and FRENCH scales in their training guide. Most articles in the literature assess indirect validity, based on a single scale, and according to patient outcome and the number of resources that were required [1, 2]. Reliability is assessed by inter-observer consistency of responses, but does not assess whether the response is correct or not [3]. Most studies evaluate only one scale [1], with users who are familiar with it and use it daily. To compare two scales, you have to test users who do not have a known scale.

The FRENCH and ESI scales are very different. Our objective was to identify the scale that would give better results on graduate nurses and student nurses with low experience.

Regarding the first point, Talebpour and Miraghi study indicates that ESI has a tendency to over-sort. However, this cited study was based only on respiratory failure patients. Our study included 120 different cases including all the symptoms of emergency department visits, proposed by ESI and FRENCH scales inventors. This may explain the differences in results.

We fully agree on the subjectivity of levels 1 and 2 in the ESI scale; and substantial clinical expertise is needed to differentiate these situations from others. This explains why the rate of correct response is lower for students than for senior nurses. Our results indicate that this uncertainty on the part of students is exerted towards subtriage, probably because they have more difficulty identifying the severity of a situation.

In our opinion, the rate of under- or over-triage for specific conditions is not comparable to the rate of under- or over-triage for all illness in our study. In fact, we would expect to see more overtriage in a group of patients with all chest pain than in patients presenting all conditions [4]. Similarly, the lower rate of correct answers among students than among experienced nurses confirms the importance of professional experience in using these tools. In order to compare two scales, it is necessary to test users who do not have a known scale. This probably explains why the kappa coefficients of agreement are lower in our study than in the meta-analysis cited [5].

We also concur with your analysis on the importance of the structure of the scenarios. For this reason, our study used the official scenarios of the ESI and FRENCH scale training guides, tested and distributed by the scale inventors. Paper scenarios obtain different results of triage compared to real cases. However it allows a better inter-individual comparability of the triage. However, paper-cases may not be representative of real clinical practice in ED and leave room for imagination. Cases simulated by an actor would not have this limitation. Furthermore, as the clinical scenarios were performed differently for the two scales, the differences observed may be due to differences in the difficulty of the scenarios (level 1–2 scenarios: 13/60 for French and 26/60 for ESI). Using the same scenarios, by consensus of experts on both scales would not have such important limitation. However, the evaluation of the clinical cases by the experts who constructed each scale seemed more robust than a comparative evaluation by independent experts.

## Data Availability

Not applicable.
